# Agreement between nicotine metabolites in blood and self-reported smoking status: The Netherlands Epidemiology of Obesity study

**DOI:** 10.1016/j.abrep.2022.100457

**Published:** 2022-09-23

**Authors:** Sofia Folpmers, Dennis O Mook-Kanamori, Renée de Mutsert, Frits R. Rosendaal, Ko Willems van Dijk, Diana van Heemst, Raymond Noordam, Saskia le Cessie

**Affiliations:** a Department of Clinical Epidemiology, Leiden University Medical Center, Leiden, the Netherlands; b Department of Public Health and Primary Care, Leiden University Medical Center, Leiden, the Netherlands; c Department of Internal Medicine, Division of Endocrinology, Leiden University Medical Center, Leiden, the Netherlands; d Einthoven Laboratory for Experimental Vascular Medicine, Leiden University Medical Center, Leiden, the Netherlands; e Department of Human Genetics, Leiden University Medical Center, Leiden, the Netherlands; f Department of Internal Medicine, Section of Gerontology and Geriatrics, Leiden University Medical Center, Leiden, the Netherlands; g Department of Biomedical Data Sciences, Leiden University Medical Center, Leiden, the Netherlands

**Keywords:** Smoking, Agreement, Nicotine metabolites, Self reported smoking status

## Abstract

**Introduction:**

Self-report and nicotine detection are methods to measure smoking exposure and can both lead to misclassification. It is important to highlight discrepancies between these two methods in the context of epidemiological research.

**Objective:**

The aim of this cross-sectional study is to assess the agreements between self-reported smoking status and nicotine metabolite detection.

**Methods:**

Data of 599 participants from the Netherlands Epidemiology of Obesity study were used to compare serum metabolite levels of five nicotine metabolites (cotinine, hydroxy-cotinine, cotinine *N*-Oxide, norcotinine, 3-hydroxy-cotinine-glucuronide) between self-reported never smokers (n = 245), former smokers (n = 283) and current smokers (n = 71). We assessed whether metabolites were absent or present and used logistic regression to discriminate between current and never smokers based on nicotine metabolite information. A classification tree was derived to classify individuals into current smokers and non/former smokers based on metabolite information.

**Results:**

In 94% of the self-reported current smokers, at least one metabolite was present, versus in 19% of the former smokers and in 10% of the never smokers. In none of the never smokers, cotinine-*n*-oxide, 3-hydroxy-cotinine-*n*-glucorinide or norcotinine was present, while at least one of these metabolites was detected in 68% of the self-reported current smokers. The classification tree classified 95% of the participants in accordance to their self-reported smoking status. All self-reported smokers who were classified as non-smokers according to the metabolite profile, had reported to be occasional smokers.

**Conclusion:**

The agreement between self-reported smoking status and metabolite information was high. This indicates that self-reported smoking status is generally reliable.

## Highlights

• Selfreported smoking status and nicotine metabolites information often agreed.
• In none of never smokers, more than two nicotine metabolites were detected.
• Two or more nicotine metabolites were present in all self-reported regular smokers.
• Metabolic information differed between self-reported regular and occasional smokers.

## 1 Introduction

Self-reported smoking status has been used most frequently as measure for smoking exposure as it is cheap and easy to collect. However, data based on self-report are error prone as participants may not fill in their smoking habits truthfully. For instance, pregnant people or individuals from households with children are sometimes reluctant to confide that they smoke (Shipton et al., 2009). Self-reported data thereby result in an underestimation of true smoking prevalence (Gorber et al., 2009).

Another method to asses smoking exposure is by measuring nicotine metabolites as biomarkers, with targeted metabolomics in for example blood (Cross et al., 2014). Nicotine’s primary metabolite product is cotinine, as 70–80 % of nicotine is metabolized to cotinine by C-oxidation, catalyzed by the CYP2A6 enzyme. Of nicotine and nicotine metabolism products, cotinine is most studied. For example, recent studies found high agreement between cotinine saliva levels and self-reported current smoking status in veterans (McGinnis et al., 2022) and positive associations between urine cotinine levels and smoking properties such as the number of cigarettes consumed per day and the time to first cigarette (Yang et al., 2020).

Cotinine has the longest half-life of 16 to 20 h in blood (Miller et al., 2010). This indicates that nicotine metabolites in blood may be specific for short-term smoking behavior. Nicotine metabolites are generally seen as a more objective representation of short-term smoking exposure than self-report (Cross et al., 2014). However metabolites assays may be influenced by environmental tobacco smoking, interindividual variations in inhalation depth and variability in detection limits (Hukkanen et al., 2005).

Both methods of measuring smoking exposure are subjective to misclassification. In order to increase reliability of studies on the effects of smoking exposure, it is necessary to quantify the agreement between self-reported and metabolomic smoking data.

The aim of this study was to determine the agreement between self-reported smoking status and the presence of multiple nicotine metabolites in blood and to develop a classification method for smoking status based on smoking metabolite data.

## 2 Material and methods

### 2.1 Study population

We used data from a subset of the Netherlands Epidemiology of Obesity (NEO) study (de Mutsert et al., 2013, Faquih et al., 2020), which is a population-based prospective cohort of 6671 individuals aged between 45 and 65 years recruited between 2008 and 2012. As part of the NEO study, all inhabitants between 45 and 65 years from the municipality of Leiderdorp were invited to participate irrespective of their BMI (n = 1671).

For the present study, metabolites were measured in a subgroup of these individuals, consisting of 599 European-ancestry participants with fasting blood sample and abdominal imaging available. Detailed information on the study design and data collection has been described previously (de Mutsert et al., 2013, Faquih et al., 2020). The study was approved by the medical ethical committee of the Leiden University Medical Centre (LUMC) and all participants provided written informed consent.

### 2.2 Data collection

Before the first study visit, participants filled in a general questionnaire on demography, health and medical history, with questions about smoking status, exposure and history. In one of the questions participants were asked to indicate if they had never smoked, formerly smoked, smoked occasionally or regularly. The last two were considered current smokers. After an overnight fast, participants were invited in the NEO study center at LUMC for a physical examination including blood sampling. Metabolic profiles were measured in 2019 in the fasting blood samples, which were stored at −80C, using Ultra-High-Performance Liquid Chromatography Mass Spectrometry (UHPLC-MS/MS). We considered five xenobiotics: cotinine, and important products of cotinine: norcotinine, hydroxy-cotinine, cotinine-*N*-glucuronide and 3-hydroxy-cotinine-glucorinide, as smoking metabolites. Measured units are ion counts as they were detected using Mass Spectrometry and therefore represent semi-quantitative values (van Waateringe et al., 2017). The Nicotine Metabolite Ratio (NMR), defined as the ratio of hydroxy-cotinine and cotinine, was calculated as a measure for nicotine metabolite speed (Dempsey et al., 2004). In this calculation, undetectable hydroxy-cotinine values were set to 0 and the NMR was undefined if cotinine was undetectable.

### 2.3 Statistical analysis

Participant characteristics and metabolites characteristics were summerized in three self-reported groups: never, former and current smokers. Continuous variables were reported with means and standard deviations, or medians and interquartile ranges for skewed distributions, and categorical variables with numbers and percentiles. We specifically reported the number of not detected measurements of the different metabolites for each group. A not detected measurement could either indicate a value below the detection limit, or complete absence of the metabolite. Per participant the number of smoking metabolites with measurements (“detected” metabolites) was counted and the numbers were compared between the never, current and former smokers’ groups. Logistic regression was used to discriminate between current and never smokers (former smokers were excluded from these analyses because they fall in between the other two categories). For each metabolite, two independent variables were used: a binary variable indicating whether a measurement of the metabolite was present (1 = present, 0 = absent) and a second variable equal to the log transformed metabolite level, with value 0 when the metabolite was not detected. Details are given in supplementary material. The results of the logistic regression were used to create a classification tree to predict smoking status based on metabolite information. Data were analyzed using R statistics version 4.0.3, packages table1, pROC and dplyr.

## 3 Results

### 3.1 General characteristics

Table 1 shows results separately for self-reported current smokers (n = 283, 12 %), former smokers (n = 283, 47 %) and those who never smoked (n = 245, 41 %). In 94 % (67/71) of the current smokers at least one metabolite was detected, versus in 19 % (55/283) of former smokers and in 10 % (25/245) of those who never smoked. In five of the never smokers only hydroxy-cotinine was present, in 18 only cotinine and in two both cotinine and hydroxy-cotinine (Suplement Table 1). When these metabolites were detectable, the median values were lower in never smokers and former smokers than smokers (Supplement Figure 1). In none of the never smokers were any of the metabolites cotinine-*n*-oxide, 3-hydroxy-cotinine-*n*-glucorinide or norcotinine detectable, while at least one of these metabolites was present in 48/71 (68 %) of current smokers. Cotinine was the metabolite most frequently present in former smokers (49/283; 17 %).

**Table 1:** Characteristics of the study population (n = 599), according to self reported smoking status, with current smokers further categorized as regular smokers and occasional smokers. (Mean (SD), medians (Q1-Q3) or frequencies (%) are presented).

	**Never smoker** (n = 245)	**Former smoker** (n = 283)	**Current smoker** **occasional** (n = 24)	**Current smoker** **Regular** **(n = 47)**
**Sex (Male)**	108 (44.1 %)	140 (49.5 %)	14 (58.3 %)	22 (46.8 %)
**Age (years)**	54.4 (6.1)	57.0 (5.6)	54.5 (5.7)	56.2 (6.3)
**BMI (kg/m2)**	25.8 (4.23)	26.1 (3.88)	25.4 (3.29)	25.2 (4.04)
**Number of packyears**	0 (0–0)	7.14 (2.90–16.1)	8.55 (2.36–19.8)	23.6 (14.3–35.7)
**Cotinine (ion counts x10e5)**	4.82 (2.80–12.0)	3.95 (1.30–10.9)	16.8 (3.49–169)	337 (60.0–2190)
Not detected	225 (91.8 %)	234 (82.7 %)	4 (16.7 %)	0 (0 %)
**Hydroxy-cotinine (ion counts x10e5)**	6.20 (5.43–7.72)	6.68 (2.21–12.7)	16.4 (5.09–22.9)	37.7 (19.6–137)
Not detected	238 (97.1 %)	265 (93.6 %)	11 (45.8 %)	0 (0 %)
**Cotinine *N*-Oxide (ion counts x10e5)**	NA	3.35 (2.69–4.50)	0.89 (0.879–1.98)	3.06 (2.15–4.82)
Not detected	245 (100 %)	279 (98.6 %)	21 (87.5 %)	6 (12.8 %)
**3-hydroxy-cotinine-glucuronide (ion counts x10e5)**	NA	2.43 (0.57–2.86)	1.08 (0.795–1.52)	1.52 (1.22–2.72)
Not detected	245 (100 %)	278 (98.2 %)	19 (79.2 %)	6 (12.8 %)
**Norcotinine (ion counts x10e5)**	NA	2.87 (2.19–3.56)	2.48 (1.86–3.38)	2.92 (2.22–3.74)
Not detected	245 (100 %)	281 (99.3 %)	21 (87.5 %)	11 (23.4 %)
**Nicotine Metabolite Ratio**	0 (0–0)	0 (0–0)	0.01 (0–0.37)	0.23 (0.01–0.56)
Not defined*	225 (91.8 %)	234 (82.7 %)	4 (16.7 %)	0 (0 %)
**Number of detected metabolites**				
0	220 (89.8 %)	228 (80.6 %)	4 (16.7 %)	0 (0 %)
1	23 (9.4 %)	43 (15.2 %)	7 (29.2 %)	0 (0 %)
2	2 (0.8 %)	7 (2.5 %)	8 (33.3 %)	4 (8.5 %)
3	0 (0.0 %)	1 (0.4 %)	2 (8.3 %)	2 (4.3 %)
4	0 (0.0 %)	2 (0.7 %)	0 (0 %)	7 (14.9 %)
5	0 (0.0 %)	2 (0.7 %)	3 (12.5 %)	34 (72.3 %)

NA:the value could not have been calculated because no individual had observed levels of the metabolite.

* NMR is not defined when cotinine level was below detection limit.

Of the current smokers, 34 % (24/71) declared to smoke occasionally. Regarding these occasional smokers, in 83 % (20/24) cotinine was detected and in 54 % (13/24) hydroxy-cotinine, while these metabolites were invariably (100 %) present in regular smokers. 91 % (43/47) of regular smokers had 3 or more metabolites detected compared with 21 % (5/24) of the occasional smokers. The current smokers without any metabolites present (4/71) all reported to be occasional smokers. Of the 23 current smokers without detectable cotinine-*n*-oxide, 3-hydroxy-cotinine-glucuronide and norcotinine, 83 % (19/23) were occasional smokers, while of the 37 smokers with all five metabolites present, only 3 were occasional smokers. The median NMR was substantially higher in current smokers but the variation was large (IQR 0.01–0.56, minimum 0.00, max 48).

### 3.2 Classification tree

Results of the logistic regression analyses can be found in the supplementary material. The results were used to develop a classification tree (Supplementary Figure 2). Three different situations are distinguished: (a) none of the five metabolites are present, in which case an individual is classified as a non-smoker, (b) at least one of the metabolites cotinine-*n*-oxide, 3-hydroxy-cotinine glucuronide or norcotinine is present, in which case an individual is classified as smoker and (c) only cotinine or hydroxy-cotinine is present. In the latter case the probability to be a smoker can be calculated using the formula given in Supplementary Figure 2, and an individual is classified to be a smoker when this probability is larger than 50 %.

### 3.3 Agreement between metabolites and self-reported smoking status

The classification tree was applied on all participants (Table 2). In 574 of 599 participants (96 %), the smoking information in metabolites agreed with the self-reported smoking information.

**Table 2:** Agreement between self-reported smoking status and smoking classification based on metabolite information, with current smokers further divided into regular and occasional smokers.

**Classification based on metabolite information**		**Never smoker**	**Former smoker**	**Current smoker** **occasional**	**Current smoker regular**
**Smoker**		2 (0.2 %)	12 (4.2 %)	13 (54.2 %)	47 (100 %)
**Non-smoker**		243 (99.2 %)	271 (95.8 %)	11 (45.8 %)	0 (0 %)

Twelve of the 283 former smokers (4 %) were classified as current smoker, five of them because 3-hydroxy-cotinine-glucuronide, norcotinine and/or cotinine-*n*-oxide was present. For 50 former smokers (18 %), only cotinine and/or hydroxy-cotinine was present, and the predicted probability to be a smoker ranged from 10 % to 87 % with 7 participants with a predicted probability higher than 50 %.

In never smokers, 10 % (25/245) had cotinine or hydroxy-cotinine detected and two of them (0.8 %) had a predicted probability higher than 50 %. For current smokers, 15 % (11/71) would be classified as a non-smoker, 4 of them because no metabolites were detected, for 7 smokers only cotinine or hydroxy-cotinine was present and the predicted probability to be a smoker was below 50 %. All 11 current smokers who were classified as non-smokers were occasional smokers.

## 4 Discussion

In this cross-sectional study of n = 599 individuals, we explored agreements between two commonly used smoking exposure methods; self-reported smoking status and metabolite detection. Logistic regression resulted in a classification tree with multiple scenarios. Over 95 % of the self-reported data were in agreement with the metabolite data.

Most studies on smoking exposure focus on linking health effects to smoking exposure, or highlight the disagreement between self-report and smoking metabolites, with faulty self-report as most probable cause of disagreement (Rebagliato, 2002). Our results are in line with previous studies that found that self-reported data on smoking can lead to underreporting of smoking habits, although in our study underreporting seems limited; fewer than 1 % of the reported never smokers were classified to be a smoker based on the metabolite information, and only 4 % of former smokers were classified as a smoker. High metabolite values in non smokers do not necessarily indicate concealing recent smoking. Environmental tobacco smoke or household smoking could cause the detection of these xenobiotics (Yang et al., 2022, Onoue et al., 2022). However, concentrations of metabolites due to environmental tobacco smoke in blood were found to be generally lower than the concentrations at which we classified individuals to be smokers (Hukkanen et al., 2005).

It is noticeable that the smoking metabolites cotinine-*n*-oxide, 3-hydroxy cotinine glucuronide and norcotinine were undetectable in all never smokers and that using these metabolites increased the specificity of the classification to 100 %. These results are in line with results of van Waateringe et al. (2017), who found high test sensitivities for cotinine in plasma and urine and cotinine-*n*-oxide in urine. However, defining smoking exposure based on these metabolites is not perfect as the metabolites cotinine-*n*-oxide, 3-hydroxy-cotinine-glucuronide and norcotinine were not present in all smokers, and in a small subgroup of smokers, no metabolites could be detected at all. In line, these individuals all reported to be occasional smokers. Potential explanations may be that some occasional smokers did not smoke shortly before blood draw, as nicotine biomerkers in general reflect short term exposure to tobacco smoke, or that their smoking habits differ, as smoking habits have been shown to significantly impact biomarker levels (Shenker et al., 2013, Yang et al., 2021). Occasional smokers had on average fewer metabolites detected compared to current smokers and all current smokers who were classified based on the metabolites as non-smokers reported to be occasional smokers. This suggests that reseachers should use more refined smoking categories and distinguish between regular and occasional smokers instead of just chotomising people into current, former and non smokers.

This study has strengths and limitations. The focus on (dis)agreements between self-report and nicotine metabolite detection is a strength, as the aim is to highlight discrepancies between the two methods in an example dataset as proof of principle. We do not address any links with other outcome variables or diseases. A limitation is that we did not collect information on environmental exposure to smoking, as this could increase metabolite levels in blood. Furthermore no definitions of current, occasional, and regular smoking were provided in the questionnaire, which may have affect concordance with metabolites. Another weakness is that we have not validated the classification method in an independent dataset. Included participants were between 45 and 65 years old and of European ancestry, which may lower generalizability to other age groups and ethnicities.

In conclusion, agreement between smoking metabolite information and self reported smoking status is high. Reseachers should, when possible, distinguish between people who report to be regular smokers and occasional smokers.

Statements and Declarations.

**Ethical approval**.

The Netherlands Epidemiology of Obesity study was approved by the medical ethical committee of the Leiden University Medical Centre (LUMC). All participants provided written informed consent.

## Funding

Part of this work was supported by the VELUX Stiftung [Grant No 1165] to DvH and RN.

## CRediT authorship contribution statement

**Sofia Folpmers:** Formal analysis, Writing – original draft. **Dennis O Mook-Kanamori:** Conceptualization, Investigation, Writing – review & editing. **Renée de Mutsert:** Conceptualization, Investigation, Writing – review & editing, Supervision. **Frits R. Rosendaal:** Conceptualization, Writing – review & editing, Supervision. **Ko Willems van Dijk:** Investigation, Writing – review & editing. **Diana van Heemst:** Writing – review & editing. **Raymond Noordam:** Conceptualization, Investigation, Writing – review & editing. **Saskia le Cessie:** Conceptualization, Methodology, Formal analysis, Writing – original draft, Writing – review & editing.

## Declaration of Competing Interest

Dennis O Mook-Kanamori reports a relationship with Metabolon INC that includes: employment. The other authors declare that they have no known competing financial interests or personal relationships that could have appeared to influence the work reported in this paper.
