# Supplementary material for: Agreement between nicotine metabolites in blood and self-reported smoking status: The Netherlands Epidemiology of Obesity study
Source: Addict Behav Rep. 2022 Sep 23;16:100457. doi: 10.1016/j.abrep.2022.100457 (PMC9519471; doi:10.1016/j.abrep.2022.100457)
Supplement: Supplementary data 1 [file mmc1.docx]

Supplementary Information

**Agreement between nicotine metabolites in blood and self-reported smoking status: the Netherlands Epidemiology of Obesity study**

**Sofia Folpmers^1^** • **Dennis O Mook-Kanamori^1,2^** • **Renée de Mutsert^1^** • **Frits R. Rosendaal^1^** • **Ko Willems van Dijk^3,4,5^** • **Diana van Heemst^6^** • **Raymond Noordam^6^** • **Saskia Le Cessie^1,7^**

Contents

[Supplementary Table 1 2](#_Toc111465421)

[Supplementary Figure 1 2](#_Toc111465422)

[Supplementary Figure 2 4](#_Toc111465423)

[Details of the performed logistic regression analysis 5](#_Toc111465424)

# Supplementary Table 1

Supplementary Table 1. Relation between detectable values of cotinine and hydroxy-cotinine.

|  |  | **Hydroxy-cotinine** | |
| --- | --- | --- | --- |
|  | **Cotinine** | Detected | Not detected |
| **Never smokers** | Detected | 2 | 18 |
|  | Not detected | 5 | 220 |
|  |  |  |  |
| **Former Smokers** | Detected | 12 | 37 |
|  | Not detected | 6 | 228 |
|  |  |  |  |
| **Current Smokers** | Detected | 60 | 7 |
|  | Not detected | 0 | 4 |

# Supplementary Figure 1

**
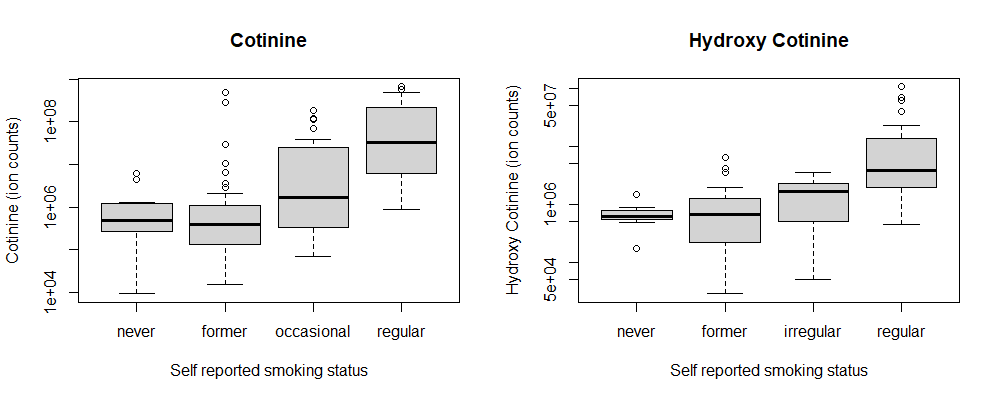
**

**Supplementary Figure 1** Distribution of cotinine and hydroxy cotinine in the smokers (regular and occasional), former smokers and never smokers, in the subgroup in which these metabolites were detectable. Number of undetected values for cotinine were 225/245 (92%) in never smokers, 234/283 (83%) in former smokers, 4/24 (17%) in occasional smokers and 0/47(0%) in regular smokers; for hydroxy cotinine the numbers were 238/245 (97%), 265/283 (94%) and 11/24 (46%) and 0/47 (0%), respectively.

# Supplementary Figure 2

**Supplementary Figure 2** Classification tree for smoking versus non-smoking obtained using a multiple logistic regression model. The probabilty to be a smoker for those with only cotinine and/or hydroxy cotinine present is equal to Pr(smoker) = exp(Z)/(1+exp(Z)), with Z = -4.147+0.315 [cotinine present]+1.695 [hydroxy cotinine present]+0.230 ln(cotinine value)+0.021 ln(hydroxy cotinine value).

# Details of the performed logistic regression analysis

Logistic regression was used to discriminate between current and never smokers (former smokers were excluded). We intended to use two independent variables for each metabolite: a binary variable indicating whether levels of the metabolite were detected (1= present, 0 = absent) and a second variable equal to the log transformed metabolite level if present and 0 otherwise. In a logistic model with these two independent variables , the constant term (intercept) equals the predicted percentage of smokers (on log-odds scale) when the metabolite measurement is missing, while the other two regression coefficients model the relation between metabolite value and smoking probability when a metabolite value is present.

Metabolites cotinine-n-oxide, 3-hydroxy-cotinine-n-glucorinide and norcotinine were not detected in any of the never smokers. This will yield infinite regression coefficients in a logistic regression model as individuals with any of these metabolites present will have a predicted probability of 1 to be a smoker.

We therefore fitted a logistic model excluding individuals with cotinine-n-oxide, 3-hydroxy-cotinine-n-glucorinide and/or norcotinine levels present. As independent variables were used: levels of cotinine (natural log transformed) and hydroxy cotinine (natural log transformed) and two indicator variables indicating whether cotinine levels and hydroxy cotinine levels were detected. Supplementary Table 1 shows the regression coefficients for this model.

Supplementary Table 1. Coefficients and standard errors of the fitted logistic regression model to discriminate between current smokers and never smokers, excluding individuals with cotinine-n-oxide, 3-hydroxy-cotinine-n-glucorinide and/or norcotinine levels present.

| variable | Coefficient | Standard error |
| --- | --- | --- |
| (Intercept) | -4.15 | 0.51 |
| Cotine present (yes/no) | 0.31 | 3.02 |
| Hydroxy Cotine present (yes/no) | 1.70 | 5.97 |
| Ln(cotinine) | 0.23 | 0.22 |
| Ln(hydroxy cotinine) | 0.021 | 0.45 |

The predicted probability to be a smoker if both cotinine and hydroxy cotinine were not present, was 1.8%. The Area Under the Curve of this regression model was: 0.96 (95% CI: 0.93-0.99) (supplementary Figure 3)


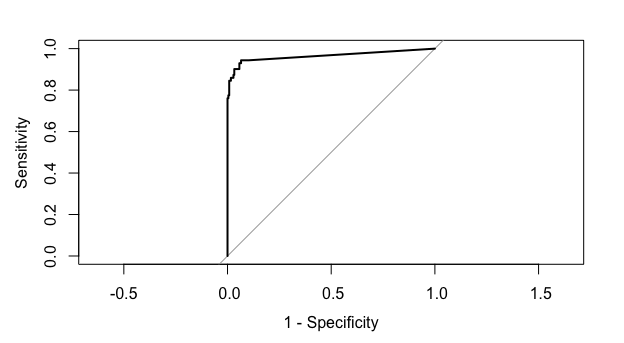


Suplementary Figure 3: ROC plot corresponding to the logistic regression model descriminating between current smokers and never smokers using a binary variable (1=present, 0 = absent) and a second variable equal to the log transformed metabolite level if present and 0 otherwise for both cotinine and hydroxy cotinine.
